# Electrospun Nanofibers Revisited: An Update on the Emerging Applications in Nanomedicine

**DOI:** 10.3390/ma15051934

**Published:** 2022-03-04

**Authors:** Nehal E. Elsadek, Abdalrazeq Nagah, Tarek M. Ibrahim, Hitesh Chopra, Ghada A. Ghonaim, Sherif E. Emam, Simona Cavalu, Mohamed S. Attia

**Affiliations:** 1 Department of Pharmacokinetics and Biopharmaceutics, Institute of Biomedical Sciences, Tokushima University, 1-78-1 Sho-machi, Tokushima 770-8505, Japan; nehal.emam92@yahoo.com; 2 Faculty of Pharmacy, Zagazig University, Zagazig 44519, Egypt; abdalrazeqnagah@gmail.com (A.N.); ghada99mahmoud@gmail.com (G.A.G.); 3 Department of Pharmaceutics, Faculty of Pharmacy, Zagazig University, Zagazig 44519, Egypt; tarekmetwally333@gmail.com (T.M.I.); sherif.emam86@yahoo.com (S.E.E.); 4 Chitkara College of Pharmacy, Chitkara University, Punjab 140401, India; chopraontheride@gmail.com; 5 Faculty of Medicine and Pharmacy, University of Oradea, P-ta 1 Decembrie 10, 410087 Oradea, Romania

**Keywords:** electrospinning, nanofibers, implants, wound healing, nanomedicine, targeted delivery, biopolymers

## Abstract

Electrospinning (ES) has become a straightforward and customizable drug delivery technique for fabricating drug-loaded nanofibers (NFs) using various biodegradable and non-biodegradable polymers. One of NF’s pros is to provide a controlled drug release through managing the NF structure by changing the spinneret type and nature of the used polymer. Electrospun NFs are employed as implants in several applications including, cancer therapy, microbial infections, and regenerative medicine. These implants facilitate a unique local delivery of chemotherapy because of their high loading capability, wide surface area, and cost-effectiveness. Multi-drug combination, magnetic, thermal, and gene therapies are promising strategies for improving chemotherapeutic efficiency. In addition, implants are recognized as an effective antimicrobial drug delivery system overriding drawbacks of traditional antibiotic administration routes such as their bioavailability and dosage levels. Recently, a sophisticated strategy has emerged for wound healing by producing biomimetic nanofibrous materials with clinically relevant properties and desirable loading capability with regenerative agents. Electrospun NFs have proposed unique solutions, including pelvic organ prolapse treatment, viable alternatives to surgical operations, and dental tissue regeneration. Conventional ES setups include difficult-assembled mega-sized equipment producing bulky matrices with inadequate stability and storage. Lately, there has become an increasing need for portable ES devices using completely available off-shelf materials to yield highly-efficient NFs for dressing wounds and rapid hemostasis. This review covers recent updates on electrospun NFs in nanomedicine applications. ES of biopolymers and drugs is discussed regarding their current scope and future outlook.

## 1. Introduction

Electrospinning (ES) is an old concept for fiber fabrication introduced in 1897 by Rayleigh and Kundu [1]. It aims at producing ultrafine non-woven nanofibers (NFs) by utilizing the action of high voltage with low current on the shape of the electroconductive liquid in a polymeric solution. The polymeric solution is pumped through a spinneret or syringe nozzle while voltage is applied between the spinneret and a collector; hence, it causes the surface of the liquid to form a cone (Taylor cone) with a rounded tip. This mechanism can be attributed to electrostatic repulsion on a droplet. When wiring electricity in the spinneret setup, charges start to build upon the droplet’s surface, creating an electrostatic repulsion of similar magnitude to the surface tension and causing it to stretch and form the Taylor cone [2]. When the voltage exceeds a certain threshold, the electrostatic repulsion overcomes the surface tension causing the rounded tip to emit a jet of charged liquid floating in the space between the spinneret and the collector surface. Eventually, the solvent evaporates, leaving the desired product on the collector [1,3].

The ultrathin NFs have multiple applications in various fields [4], such as cosmetics [5], active food packaging [6], fabricated fabricating nanofibrous sensors [7], textiles [8], and in particular, their applications in drug delivery [9,10,11], wound healing [12,13], nanomedicine and tissue engineering [14,15].

**Figure 1:**
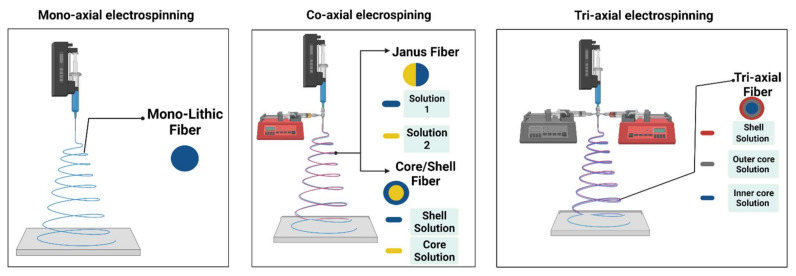
Techniques for nanofiber production.

The emerging role of ES in drug delivery systems offers a safe and easy encapsulation technique for the therapeutic cargos, maintains their structural integrity and biological activity, and accordingly, ensures a safe release at the target site [16]. At the same time, it provides a cost-effective procedure starting from fiber fabrication to the formulation of the final pharmaceutical product [17]. In addition, ES can be utilized in loading one drug alone or multiple drugs, depending on the characters of the nozzle setup. Loading one drug alone is simple and uses a one-nozzle setup which is not the case with numerous and sometimes incompatible drugs as it requires the use of other ES setups such as the coaxial nozzle setup that provides a core-shell NF, controlling the releasing kinetics of the loaded drugs, see Figure 1 [18,19]. Their dynamic ability to tailor their characters and match every drug requirement gives electrospun NFs an edge over other delivery systems. A tailored design for the NF’s diameter, porosity, and thickness is achieved by adjusting solution parameters, environmental conditions, and ES setup parameters to the point that makes the system efficient enough for its intended release mode [20,21], which can be summed up into fast, sustained, and combined or customized release.

Electrospun NFs have been the research subject to develop fibrous scaffolds that allow skin regeneration, primarily because of their compatibility with the skin’s extracellular matrix (ECM) and biodegradability. Moreover, they can provide enhanced healing rates and be loaded with a bactericidal agent to prevent tissue infection and bioactive molecules and help the regeneration process [22]. Skin wounds are generally associated with increasingly high morbidity and mortality rates that can be regarded as a matter of life and death. Despite the availability of different treatment options and delivery systems, they utterly fail to recover normal tissue states and structures, exacerbating wound infection and tissue necrosis. Tissue-engineered scaffolds aim to regenerate diseased or damaged tissue by using fabricated scaffolds that mimic the complex human tissue’s ECM. Fabricated scaffolds are designed to be porous and permit nutrient diffusion and cell migration while providing slow and steady degradation rates so that the natural ECM completely replaces it as the tissue recovers [14,15].

Recently, the emergence of single, handheld, and easy-to-use portable ES devices, has revolutionized the production of electrospun scaffolds. A portable device can produce personalized fiber matrices tailored for each case where the wounded skin tissue acts as the collector of the fiber matrices [23], in a process that reduces the usual pain associated with conventional wound dressings application or removal and enhances the patient’s compliance to treatment [24].

In this regard, this review seeks to provide an overview of nanofiber-based drug delivery and its related applications in nanomedicine on the most recent literature research. Searching literature was through Reaxys database using the keywords “Implant”, “Drug delivery”, “electrospun”, “nanofiber”, and “wound healing” interchangeably. The recent literature (last five years) has been considered relevant, but discussions were also addressed to earlier reports in this field.

## 2. Electrospun NF Implants

Advances in biomedical sciences and the increasing complexity of diagnosis and therapeutic processes necessitate the development of a unique drug delivery system to improve the bioavailability of pharmaceuticals reduce their adverse effects. Nanotechnology makes these advancements possible, which allows for the creation and design of nanoparticle-based drug delivery systems. These systems can then be improved using nanoengineered implants, which ensure controlled and sustainable release, reduce side effects, and increase the drug’s bioavailability at the target site.

ES technology has gained great attention for its potential biomedical applications since electrospun NFs are becoming more applied in treating various conditions like inflammations, neoplasia, infections, and many other diseases. Electrospun nanofibrous implants represent an ideal pharmaceutical option for the sustained release of medications in the body, which one of its roles is to improve the bioavailability of the used drug. Electrospun NFs offer a perfect therapeutic choice for various conditions as they provide a stable drug-release level in addition to the extended duration and controllable dosage [25].

### 2.1. Electrospun NF Implants for Cancer Therapy

Cancer is one of the leading causes of mortality worldwide, and cancer therapy still has a narrow margin of safety and efficacy since it affects healthy cells. Following diagnostic investigations, therapeutic techniques, and technology progress, the number of cancer survivors will increase while enhancing their quality of life by delivering more effective and efficient treatment regimens. Radiation therapy, chemotherapy, and surgical procedures are among the current cancer treatment options. Current therapy plans are insufficient and have numerous drawbacks [26,27].

Anticancer drugs are no exception since they have drawbacks such as high toxicity for normal cells, cancer therapy failure, or limited dissolvability, thus reducing their bioavailability and effectiveness [28,29]. According to the previous limitations, new therapeutic approaches and technologies become necessary to advance cancer treatment with minimum damage to normal healthy cells. Nanotechnology poses itself as a promising therapeutic approach since it provides novel treatment approaches. Electrospun NFs offer a unique drug delivery system in cancer therapy because they have high loading potential, large surface area, and are cost-effective and reliable [30].

Cancer patient survival rates are low due to some variables, including late-stage detection, the toxicity of anticancer treatment, and the poor response of many cancer types to therapy [31,32]. Targeted therapeutic approaches have been investigated in research and clinical practice to reduce the harmful side effects of conventional cancer therapies while maximizing the desired therapeutic benefits [33]. Nanotechnology provides two main therapeutic options. The first one involves encapsulating the drug with nanoparticles and targeting a specific site with this compound using a positive or a negative targeting system [34]. The second strategy entails implanting a drug-loaded system at the neoplasia site to locally deliver the required drug dosage to limit injury to normal tissues [34].

Electrospun NFs have a high surface-to-volume ratio, which improves the drug’s solubility in an aqueous environment and enhances its efficacy [35]. In addition, electrospun NF implants, in particular, can be used for localized cancer treatment, which is one of the most effective and safe treatment strategies. ES has been proven to be one of the most competitive nanotechnologies since it allows for the mass production of NFs at reasonable costs. Moreover, electrospun NFs are very promising as drug carriers, particularly for local delivery of chemotherapy [31].

**Figure 2:**
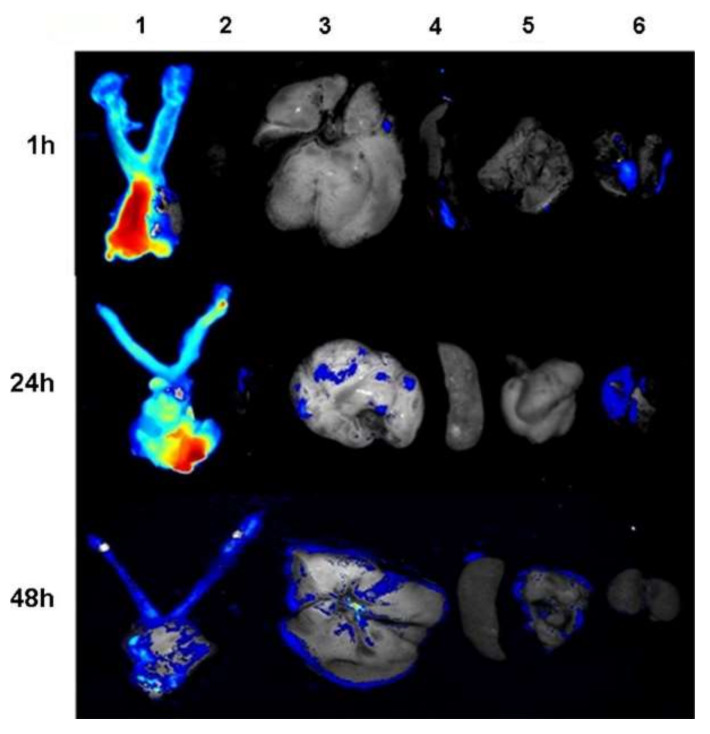
Ex vivo imaging of collected organs (1 tumor, 2 hearts, 3 livers, 4 spleens, 5 lungs and 6 kidneys) at 1, 24 and 48 h following the implantation of electrospun nanofibers into the mice’s vaginas. Reprinted with permission from ref. [36].

Extensive research has been done to study electrospun NF implants’ efficacy and safety in cancer therapy. Zong et al. [36] examined the potential role of fibrous implants in treating cervix cancer. They employed cisplatin (CIS)-loaded poly(ethylene oxide)/poly(lactide) (PLA) composite NFs for vaginal implantation to deliver CIS to cervical cancer cells (U14) in mice. In comparison to intravenous injection, vaginal implantation significantly induced more CIS accumulation in the vaginal and cervix regions than in the kidney, liver, or blood (Figure 2).

Furthermore, Liu et al. [37] have studied the role of electrospun NFs in treating secondary hepatic carcinoma (SHCC). After explorative laparotomy in mice, they applied doxorubicin-loaded PLA (DOX-PLA) electrospun NFs as implants. Results showed an increased survival of the affected mice from 14 days to 38 days, in addition to a remarkable inhibition of the SHCC. These significant effects hold the promise of treating SHCC in vivo [37]. Similarly, another group of researchers has applied dacarbazine-loaded poly(vinyl alcohol) (PVA) electrospun NFs as brain implants to treat glioblastoma [38]. This system showed high encapsulation efficiency (>80%) and controlled dacarbazine release, thus significantly improving its anti-tumor effects, such as apoptosis-mediated cell death and DNA damage.

One promising approach that has been created through coaxial electrospinning is core-shell structured electrospun NFs, which allow more sustained drug release by inhibiting the initial burst release. Two different drugs can be encapsulated simultaneously using core-shell electrospun NFs: one in the core and the other in the shell, and released at varying rates depending on the drug nature and polymers utilized and the degree of degradability of the outer layer [39].

**Figure 3:**
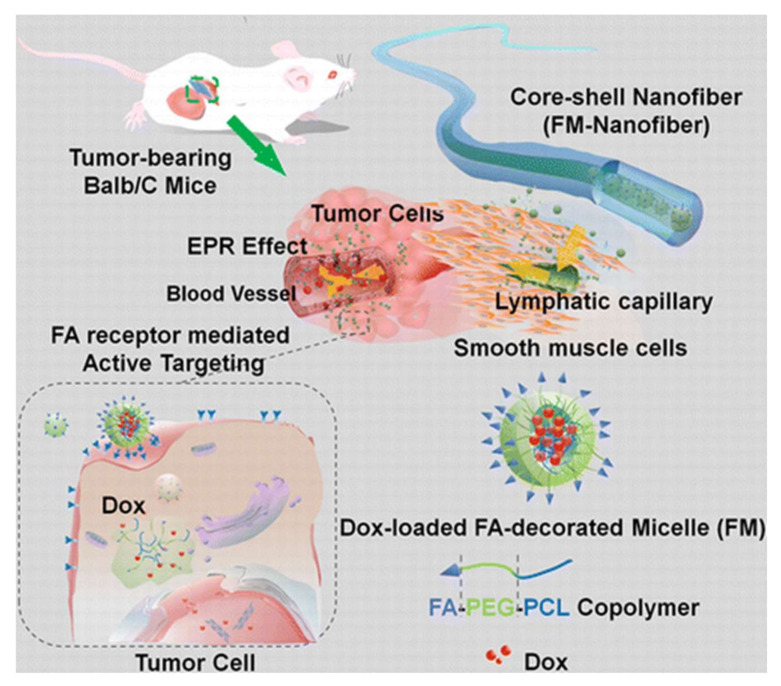
Coaxial electrospinning-prepared implantable Doxorubicin-loaded micelles in NFs for effective cancer therapy. Reprinted with permission from ref. [40].

The core-shell NFs system introduced by Yang et al. [40] has increased nanoparticle stability, such as micelles. They developed an implantable drug delivery system for the localized delivery of anticancer drug through the coaxial ES of PVA/active targeting DOX-encapsulating micelles (composed through the self-assembly of folate-conjugated poly(ε-caprolactone) (PCL)/poly(ethylene glycol) (PEG) copolymer as the core and cross-linked gelatin as the shell (Figure 3). The degradation of NF matrix causes these active-targeting micelles to be released from the devices in a time-dependent fashion, quickly accumulating around the tumor tissue via interstitial transport and the enhanced permeation and retention (EPR) effect, lowering the dosage of the chemotherapeutic agent, frequency of administration, and adverse effects.

Multilayering ES is another approach developed to gain more control of the therapeutic agent’s discharge kinetics in the core-shell electrospun NFs, in which additional layers with different diffusion pathways and degradation behaviors are included to stop the first burst release and provide sustained release as well. Falde et al. used this approach to create an implantable multilayered 3D electrospun NF mat for the localized delivery of the chemotherapeutic agent SN-38. The system was constructed of a first barrier mesh, a second drug-loaded layer and a third barrier mesh. The in vitro release studies showed the system’s success in reducing the burst release of the drug by ten times and the sustained release of SN-38 for more than 30 days. Furthermore, the developed system was cytotoxic to Lewis Lung Carcinoma (LLC) cells for more than 20 days [41].

The use of single chemotherapy is uncommon due to the toxicity of chemotherapeutic agents at high doses and the risk of developing resistance if single chemotherapy is used for a long time. As a result, multidrug-loaded electrospun NFs are one of the most promising options for overcoming the adverse effects of single therapy, lowering the applied dose and improving chemotherapy effectiveness [42]. Table 1 summarizes some of the reported implanted multidrug-loaded electrospun NFs for localized chemotherapeutic agent delivery into the tumor.

Combination therapy is another recently adopted cancer therapy technique, in which two therapeutic strategies are integrated into one platform to enhance the antitumor effect while avoiding the negative side effects of monotherapy. Hyperthermia is one of the used approaches for targeted cancer therapy, with malignant cells being more thermosensitive than healthy ones. Using blend ES, Zhao and coworkers co-loaded DOX and MoS2, an effective photothermal agent, into chitosan/PVA electrospun NF mat to combine the photo hyperthermia and chemotherapy [66]. The heat generated from the near-infrared irradiation of 808 nm laser could sensitize the chemotherapeutic efficacy of DOX via controlling its discharge rate. The combined therapy efficiently boosted cytotoxicity against HT29 cells in vitro and in vivo, where surgical implantation of the nanofibrous mat into the tumor site could completely inhibit cancer recurrence. Similarly, another group combined the chemotherapeutic DOX with photothermal treatment: Cu9S5 through incorporating DOX-loaded Cu9S5@mSiO2 nanoparticles into PCL/gelatin nanofibrous mat. Under 980 nm laser irradiation, the combined chemo/photothermal therapy showed a more efficient tumour-suppressive effect in vivo than either single photothermal or chemo treatment [67].

Magnetic thermal therapy is another protocol that is used as a co-therapy with chemotherapy, in which iron oxide (Fe_3_O_4_) magnetic nanoparticles (MNPs) are applied as a heat source under an alternating medical field (AMF) [68]. In a similar way to photothermal gents, the magnetic thermal agents can synergize the antitumor effect of the chemotherapeutic agent that is co-loaded with them in the nanofibrous system. In a related study, Niiyama et al. loaded paclitaxel and MNPS into PCL polymer using the ES technique. The AMF irradiation could provide a sustained release of paclitaxel over six weeks. In vivo, the locally created implantable NF system showed a superior anticancer impact against the human lung (NCI-H23) cancer cell line than chemotherapy or thermal therapy alone while using an AMF of 166 kHz and 192 A [69].

Gene therapy is also one of the most widely used approaches for cancer treatment that is targeted, safe and successful. Electrospun NFs are one of the nanotechnology techniques used as a platform for cancer gene therapy delivery. They are also employed for the dual delivery of gene therapy and chemotherapy, resulting in a super additive antiproliferative effect [70]. In a related study, for the treatment of liver cancer, Che et al. fabricated nanoparticles based on the electrostatic interaction between the tumor suppressor miRNA-145, as anticancer gene therapy and cross-linked branched polyethyleneimine. The paclitaxel/PCL NFs were then coated with these nanoparticles. Upon applying the devised technology, both therapeutic compounds were released sustainably. Moreover, the concomitant delivery of paclitaxel and miRNA showed synergistic antiproliferative efficacy against hepatocellular carcinoma [71].

Nanotechnology is a highly active field as it has been evolving rapidly and becoming more and more reliable in clinical practice [72]. Electrospun NFs hold extraordinary promise to represent a breakthrough in cancer therapy as they show high loading capacity, large surface area, cost-effective production and direct delivery of various treatments. Moreover, those electrospun NFs represent a highly accurate diagnosis method such as ultra-sensitive sensing systems for cancer detection and migrating cancer cell targeting. However, electrospun NFs still need further research before introducing them to clinical practice and the market [31].

### 2.2. Electrospun NF Implants as Antimicrobial Agents

Antimicrobial agents work against bacteria and other microorganisms by preventing wall synthesis or limiting microbial development. Traditional antibiotic administration routes, such as topical and systemic administration, have some limitations in terms of bioavailability and dosage levels. Furthermore, poor antimicrobial agent distribution may reduce treatment efficacy in addition to its local and systemic side effects, such as inflammation and decreased normal gut microbiota. As a result, developing an effective antimicrobial drug delivery method is critical [73,74,75].

Electrospun NF implants are being studied for their use as novel delivery systems for antimicrobial agents using tetracycline hydrochloride with PLA, poly (ethylene-co-vinyl acetate) (PEVA) or a mixture of both [76].

Research showed that electrospun PCL/poly(trimethylene-carbonate) (PTMC) ultrafine composite fiber mats perform as drug carriers that enclose the herbal antibacterial agent: Shikonin (SKN), a highly liposoluble naphthoquinone pigment isolated from the roots of lithospermum erythrorhizon. Drug release is then controlled by PCL/PTMC blend ratio in addition to drug-loading concentration. Those mats have free radical scavenging effects in addition to the antibacterial ones. Therefore, they also represent a compelling choice for treating dermal bacterial infections or wound healing [77].

Treating infections caused by Methicillin-resistant Staphylococcus aureus (MRSA) represents a serious challenge in the healthcare field as it requires accuracy in delivering the appropriate antibiotic at the correct dose to assure efficacy. Researchers developed a biodegradable localized delivery system for combining fusidic acid, sodium fusidic and rifampicin into PLGA polymer using the ES method for creating antimicrobial drug-loaded mats. Results showed that this delivery system was effective against Staphylococcus epidermidis and two MRSA strains [78]. These drug-loaded mats were proven to be an effective therapeutic option for implant-related infections that would otherwise necessitate orthopaedic surgery; thus, they would let patients avoid surgery wherever possible.

**Figure 4:**
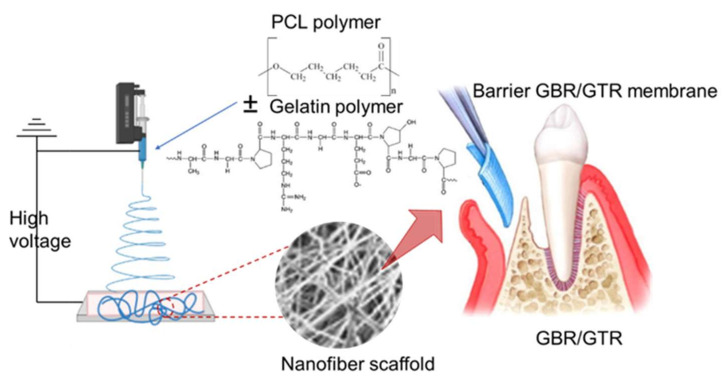
Development of a barrier membrane using the ES technique for the localized delivery of antibacterial agent in GBR/GTR applications.

Guided bone regeneration and guided tissue regeneration (GBR/GTR) have become standard techniques in bone/tissue regeneration therapy to enhance bone/tissue regeneration through the application of GBR/GTR membranes that act as a barrier for the epithelial migration into the defective site and increase cell proliferation and attachment into the defect location [79]. In clinical applications, infections can be blamed for most GBR/GTR failure cases [80]. Accordingly, developing an anti-infective GBR-GTR membrane is a practical approach to solve this burden. By loading metronidazole into PCL/gelatin NF scaffolds, Xue and coworkers developed an efficient anti-infective GBR-GTR membrane with antibacterial activity delivered locally using the ES technique (Figure 4). The metronidazole release rate depended on the loaded content in the scaffold and the presence of gelatin in the membrane [81].

Kataria et al. [82] had studied electrospun composite NFs transdermal patches for wound healing. Their research concluded that electrospun NF of sodium alginate (NaAlg)/PVA—ciprofloxacin-based transdermal patches were greatly beneficial for the local and fast delivery of medications to limit and control infections. These patches can successfully provide an efficient and fast drug delivery system to control infections. These patches show sustained drug release levels following the Korsmeyer-Peppas and Higuchi model.

### 2.3. Electrospun NF Implants Used in Regenerative Medicine

#### 2.3.1. Wound Healing

Skin is the largest organ of the human body [83]. It represents the first barrier blocking pathogens from entering the body and limiting water loss [84,85]. Acute wounds (such as surgical or traumatic wounds, as well as burns and abrasions) and chronic wounds (which do not show a normal and correctly sequenced repair) are the two main types of skin wounds. These wounds are common in diabetic or decubitus ulcers [86]. According to National Center for Health Research and Statistics, trauma cases exceed 40 million in the United States each year, costing more than USD 670 billion. Chronic wounds affect approximately 6.5 million people in the United States, with treatment costs totaling more than USD 25 billion [87]. The situation is even worse in developing countries [88,89].

There are several classification systems for skin wounds, based on the size, depth and tissues involved, as well as the cause, such as burns, traumas or chronic illnesses. Wound dressings are essential for preventing infections and ensuring a healthy environment for wound healing. Sometimes, wounds may need dermal substitutes to trigger cells migration to the afflicted location [90,91]. Full-thickness wounds are more difficult to cure than superficial wounds because they require artificial skin substitutes or autografts, as well as a healthy environment [92]. The majority of wound healing materials cannot imitate the cutaneous extracellular matrix (ECM) [93]. On the other hand, ES technology has created a unique and sophisticated way for wound healing since it can produce biomimetic nanofibrous materials from various natural and synthetic polymers with clinically relevant properties [94]. Electrospun NFs made from type I and III collagen, which are the most common collagens in the skin, are now possible thanks to advances in nanotechnology and nanoengineering [95].

Electrospun NF implants are also an effective and efficient way to treat wounds as they feature ease of application and the great potential of loading them with different pharmaceutical agents such as antibiotics or regenerative agents [96].as shown in Table 2.

Electrospun NF implants represent an excellent choice for regulating skin cell behavior through drug loading or using transmembrane receptors or intracellular signaling pathways [104]. Electrospun NFs encapsulated by type I collagen, laminin and integrin ligands prompted normal epidermal keratinocyte adhesion in humans, as well as a spreading morphology in 50% of proliferating cells, according to a study conducted by Rho and coworkers [105]. Furthermore, the effects of PCL-blended collagen nanofibrous membranes were investigated, and it was discovered that this membrane stimulates the adhesion and proliferation of human dermal fibroblasts [106].

Skin grafts are still one of the most popular and reliable methods for skin replacement or restoration because skin substitutes currently lack many key characteristics that allow them to mimic normal skin in terms of architecture and composition [107].

**Figure 5:**
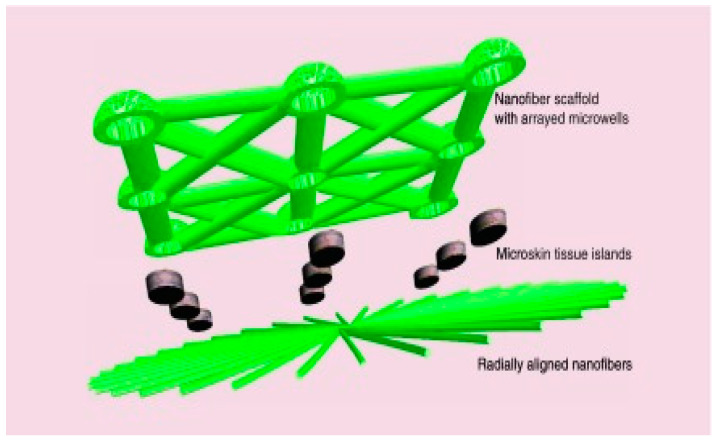
Sandwich-type NF skin grafts. Reprinted with permission from ref. [104].

Ma et al. developed a novel method combining tissue engineering technologies with clinically viable techniques such as autologous skin grafting. This method involved creating a multifunctional NF skin graft in the shape of a sandwich (Figure 5) [108].

This NF implant has several functions, including guiding cell migration to ensure proper and organized cell localization and accelerating the healing process in general by providing a higher expansion ratio and a sustained and stable release of antibiotics, which inhibits infections locally and limits antibiotic overuse toxicity.

This method was tested by implanting these sandwich-type scaffolds on skin excision in rats. Results showed a good acceptance of the scaffold in the wound site in all transplanted microskin tissue islands with a consistent distribution seven days after the surgery. The wound was completely closed 21 days after surgery, thanks to re-epithelialization via microskin grafts. This procedure will aid in healing severe burns and treating chronic injuries.

#### 2.3.2. Dental Applications

**Figure 6:**
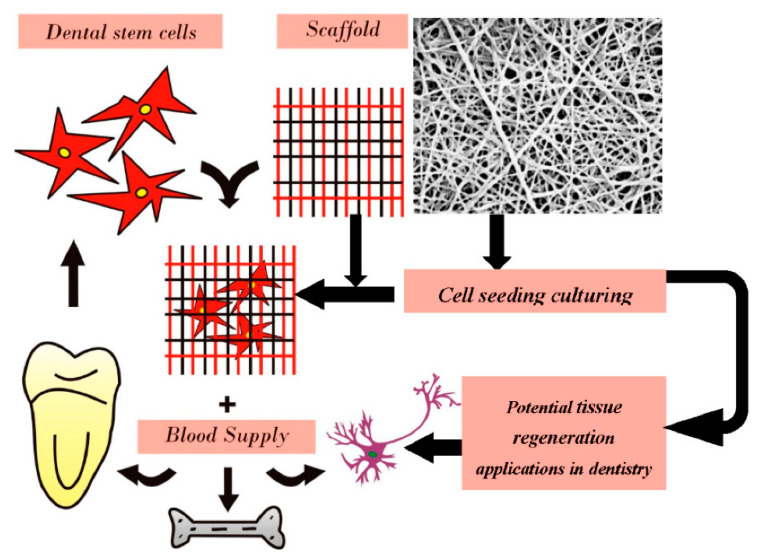
Schematic presentation for implementing electrospun NF implants in dental tissue regeneration. Republished under permission from ref. [109].

One of the major applications of electrospun NF implants is in the dental field, as they provide an excellent option for replacing dental tissues. Figure 6 shows the standard approach for utilizing electrospun NFs implants for dental tissue regeneration [109].

Numerous conditions such as dental caries and traumatic accidents may lead to losing dental tissues. Conventional treatment methods involve using different restorative materials such as ferric sulphate, hydroxide and mineral trioxide aggregates. However, these conventional methods may still result in internal resorption of teeth [110,111]. This is where electrospun NF implants make the difference as they were studied in order to assess their potential in dental regeneration. Kim e al. had created electrospun implants from PVA and hydroxyapatite (HA), which may have dentin regenerative features [112]. Additionally, electrospun NF meshes created with PCL have exhibited great potential for promoting odontogenic regeneration and differentiation. Evidence on that is the increased turnover of collagen I and other proteins when tested on human pulpal cells in vitro [113].

#### 2.3.3. THE Role of Electrospun NF Implants for the Treatment of Pelvic Organ Prolapse

Pelvic Organ Prolapse (POP) is a deteriorating urogynecological pelvic floor chronic illness that affects the quality of life of 50% of parous women over the age of 50 [114]. Surgical repair with transvaginal meshes is one of the most common treatments for POP. However, such meshes have been linked to significant problems such as long-term chronic inflammation and poor tissue integration [115]. Nanotechnology has proposed a potential and unique solution to this problem. Mukherjee et al. created electrospun NF poly L-lactic acid-co-poly-caprolactone (PLCL) meshes loaded with endometrial mesenchymal stromal cells (MSCs) that alter foreign body reactions through several mechanisms and evaluated these meshes in mice in their study. According to the findings, the use of electrospun NF meshes loaded with MSCs in the treatment of POP in mice resulted in a significant increase in angiogenesis, cell adhesion and immunological response after 6 weeks post implant. Anti-inflammatory genes were also expressed more frequently, which helped to control inflammation and promote healing. Those findings point to a high likelihood of a successful implant as well as a viable alternative to surgical operations [115].

#### 2.3.4. Electrospun NF Implants for Cardiac Tissue Engineering

Cardiovascular disorders are one of the leading causes of morbidity, reducing patients’ quality of life and resulting in a huge number of untimely deaths around the world. Heart transplantation is the only drastic treatment for end-stage cardiac failure currently available [116]. This treatment has several disadvantages, including the restricted availability of donated organs and the problems associated with immunosuppressing the patient after the procedure to prevent transplant rejection.

Cardiac cells are non-renewable; thus, cardiac cell therapy is critical for addressing the majority of heart diseases [117]. It has a lot of potential in terms of treating myocardial infarctions, for example. One of the novel therapeutic methods is integrating cells into 3D biodegradable scaffolds, which appears to be viable for preserving cell viability and promoting cell embedding following transplantation [118]. This is a significant advancement in the field of cardiac cell therapy, as it is a more advanced treatment strategy than simply injecting isolated cells.

Cardiac tissue cells impose a complex challenge because of its unique characteristics and compound structure. However, there is a huge potential for designing new techniques and conducting further research to develop ultimate novel methods of cardiac cell therapy. Electrospun NF scaffolds, which blend natural and synthetic polymers, are a viable therapy technique for cardiac diseases. The major goal is to combine the benefits of both sources to create the best design that provides the required elasticity and electrical properties and typical healthy ECM protein adhesion cellular ligands [119].

#### 2.3.5. Orthopaedic Applications

ES is a flexible technique that allows us to create NFs of a web-like design mimicking ECM, which significantly improves cell adhesion and integration [120,121]. This feature has been studied to assess its possible applications in the biomedical field. Hard tissues, such as bones and cartilages, are no exception since the polymers utilized in the electrospun NF implants play an essential role in guiding them to their intended purpose. PLGA, PCL, PLLA and their copolymers have been widely employed because they are biocompatible and biodegradable [122,123,124,125]. Because of its biodegradability, low cost, flexibility and good biocompatibility with osteoblasts, the FDA has approved the use of PCL polymers in durable implants [126]. On the other hand, most other polymers have limits in the orthopedic sector because they are not strong enough, hydrophobic or bioactive [127,128]. To overcome their low stiffness, researchers have proposed mixing these polymers with bioactive chemicals such as HA, bioactive glass (BG) and tricalcium phosphate.

Magnesium alloys were reported as promising candidates for bone fixation and tissue regeneration [129]. Furthermore, electrospun NFs loaded with HA and simvastatin had a dual effect through controlling degradation and triggering bone regeneration. Overall, in a study conducted by Kim’s group, magnesium alloy ES with a covering of PCL/HA/simvastatin NFs demonstrated promise as a drug delivery system and a hard-tissue engineering technology [130].

#### 2.3.6. Electrospun NFs as Antibacterial Coatings to Prevent Implant-Related Infections

The development of an infection and formation of bacterial biofilm over an implant surface are considered the major impediments to the success of orthopedic and dental biomaterials implantation that may require the reoperation of patients. Accordingly, there is a clear need to develop a tunable implant coating with excellent bactericidal properties. However, there are only a few available techniques for coating the required contaminants, and most of them lack effectiveness [131]. ES technology has shown its ability to tackle the limitations of the other technologies due to its high antibiotic loading efficiency and sustained drug release properties [132] (Table 3).

Kranthi et al. used the ES technique to incorporate HA and rifampicin nanoparticles into PCL composite NFs and then coated them into titanium bone implants to tackle related infections [133]. Such coating was effective even against MRSA. HA ceramic was demonstrated to resemble the bone material and increase the osteogenic cell activity [134]. The presence of HA nanoparticles within the PCL composite NFs was shown to reinforce the mechanical properties of the composite scaffold.

Furthermore, most of the approaches used to prevent implant-related infections are designed to release only one antibiotic, increasing the risk of antibiotic resistance development [135,136]. As a result, many researchers used the ES technique to apply combinatorial antibacterial therapy to implant coatings. Jahanmard et al. developed PLGA/PCL bilayer coating structure with a combinatorial antibacterial activity using ES technology [137]. Vancomycin and rifampicin co-delivery in a bilayer structure could provide a sustained antibacterial effect over several weeks. Hence, it could cover both early and delayed onset of infection in comparison with the single coating structure.

Similarly, Ashbaugh et al. used the same two polymeric fibers, PCL/PGLA, for the tunable delivery of different antibody combinations [138]. Rifampicin was co-loaded into the composite coating with either vancomycin, linezolid or daptomycin. In the in vivo mouse model of *Staphylococcus aureus* prosthetic joint infections, all three antibody combinations could inhibit the infection and biofilm growth over the implant entirely. It is worth noting that by adding rifampicin within the PCL layer, this PCL/PGLA composite coating allows the tunable delivery of the antibiotic combination, with rifampicin diffusing faster than the other antibiotic. Accordingly, the development of the known rifampicin resistance [139,140] during therapy can be overcome by avoiding the existence of rifampicin as a single agent during the treatment period.

ES is a promising technology that has been investigated extensively for its various biomedical applications and advantages. Electrospun NF goods, particularly electrospun NF implants, have undergone extensive research to be introduced into clinical practice. A lot of research has shown promising results for finding novel treatments to various serious medical conditions and cosmetic purposes. These conditions involved cancer therapy, regenerative medicine, sustained release of medications, wound healing and other surgical uses. However, this field has not been fully assessed and studied. We still need to conduct further research to achieve the most possible out of this amazing technology and fully integrate it into clinical practice.

#### 2.3.7. NF for Wound Healing Applications

Skin wounds are associated with increasingly high morbidity and mortality rates that can be regarded as a matter of life and death. While there are some available treatment options, they utterly fail to recover the damaged skin tissue’s normal state and structure, exacerbating wound infection and/or tissue necrosis. Electrospun NFs have been the subject of research to develop wound dressings that can allow for skin regeneration primarily because they are compatible with the skin’s ECM, improve skin healing rates, are degradable over time and can be quickly loaded with a bactericidal agent to prevent tissue infection and/or bioactive molecules and help with the regeneration process [22,147,148].

##### Water Resistance and Breathable Wound Dressings

NFs are currently one of the leading materials used for wound healing applications. The NFs can be molded as membranes supply excellent water resistance properties, and they also have a sufficient pore number on the surface, making them breathable, causing the moisture from the wound to escape [149,150,151]. However, a lower pore diameter guarantees water resistance [152,153,154].

Many researchers have tried to develop breathable and water-resistant wound dressings. Yue et al. developed a waterproof and breathable (W&B) membrane for wound healing [154]. Yue et al. electrospun the membrane with a perfusion speed of 0.05 mL/h to 10 mL/h and a voltage of 11 kV. The thymol-loaded ethanol-soluble polyurethane skin-like membranes were prepared. The membrane showed waterproofness to 17.6 cm of water and a comparatively better breathability rate. Apart from this, the membrane also showed antibacterial action against the wound.

Similarly, another group of researchers developed an asymmetric wettable gradient nanofibrous membrane, consisting of polyvinyl butyral (PVB)-polydimethylsiloxane (PDMS) as the top layer followed by a layer of PVB-PDMS/gelatin and gelatin as a hydrophilic layer [155]. The upper layer showed a better contact angle (CA) than the gelatin lower layer. The membrane showed water resistance to about 58.21 kPa with a water vapor transmission rate (WVTR) of 8.80 kg m^−2^d^−1^. Additionally, the membrane improved mesenchymal cells with the immobilization of stromal cell-derived factors to increase the wound healing rate.

The stretchable PDMS-embedded PVB fibrous membranes based on ethanol solvent system for W&B wound dressings were prepared by Guo et al. using the ES technique [156]. The presence of PDMS caused a reduction in pore size and enhanced surface hydrophobicity by four times. The membranes showed a WVTR of 8.98 kg m^−2^d^−1^. An increase in CA from 127.80° to 133.16° was observed after increasing PDMS content by 45%; however, the increase was not significant. This might be attributed to the presence of PDMS as a low surface energy agent on the fiber surface. However, due to surface roughness, the increase was not so prominent. In an experiment conducted with pure water, coffee, milk, methyl orange, methylene blue and rhodamine were dripped on the membrane surface. It was found that the sphere-like droplets were formed due to lyophobic properties. The membrane was tilted at an angle of 30 ± 5° so that drops could roll, and the drops tolled off smoothly without wetting the membranes indicating the antifouling property. However, in the case of soybean oil and petroleum ether, poor oleophobicity was observed due to the oleophilic nature of PDMS. Researchers developed hydrophobic microporous NF membrane via fluorosilane-modified silica (F-SiO_2_) nanoparticles, polyurethane and polyacrylonitrile solution [157]. The presence of flurosilane led to the hydrophobic behavior in the membrane. The formed solution was electrospun, and results showed that incorporating F-SiO_2_ could promote the hydrophobic nature of the membrane; thus, the membrane exhibited excellent waterproof and mechanical properties with good WVTR results. In addition, they can be used in the medical field for drug delivery applications. Another group developed a nanofibrous membrane using polyacrylonitrile, polyurethane and titanium dioxide. Apart from W&B resistance, the formed films also showed UV resistance due to UV531 and fluorinated acrylic copolymer [158]. At the same time, the hydrophobic coating showed superhydrophobicity and wettability with a CA of 152.1°.

##### Electrospun NFs as a Drug Delivery Carrier for Wound Healing

Electrospun NFs are the first choice for practitioners owing to their mimicking nature with ECM and low or nil adverse effects. Researchers have used natural polymers for wound healing applications either alone or combined with synthetic polymeric counterparts to increase their mechanical and physical properties. Depending on the need and the wound getting treated, the NFs can be impregnated with drugs and stem cells as indicated in Table 4.

The NFs containing the active moiety have been shown by various studies and are the primarily used approach during the past decade. Mohammadi et al. prepared electrospun CUR-loaded PCL /gum tragacanth NFs to treat diabetic rats [159]. The scaffolds also possessed antimicrobial action against various Gram-positive and Gram-negative bacteria. After 15 days of application of NFs, remarkably faster wound healing was shown. Morphologically, the wound showed proliferation of fibroblasts, deposition of collagen and formation of sweat glands and hair follicles, confirming the wound healing.

Similarly, another research group reported the formulation of CUR-based PCL NFs formed via the ES process [160]. The fibers were able to release the active ingredient for about three days in vitro. The human foreskin fibroblast cell lines used showed 70% enhancement in the bioavailability of CUR. Additionally, the fibers reduced the levels of interleukin-6 release from the in vitro model and showed an acceleration in wound closure rate in the streptozotocin-induced diabetic model. Similar membranes were prepared by ES technique using the gum tragacanth, PLGA and tetracycline as the active agent. These NFs showed smooth, beadles morphology and controlled release of tetracycline for over 75 days, with an initial burst of 19% for the first two days [161].

**Figure 7:**
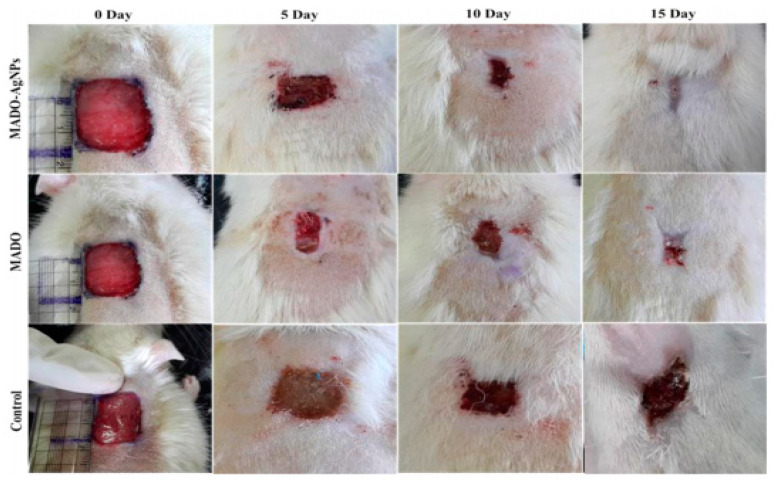
Wound appearance at 0, 5, 10 and 15 days after grafting with poly(dopamine methacrylamide-co-methyl methacrylate) (MADO)-AgNPs, MADO NF, and control [163].

Golchin et al. prepared CUR incorporated CS/PVA/Carbopol/PCL nanofibrous composite to deliver buccal fat pad-derived mesenchymal cells and CUR [162]. The formed nanofibrous allowed cell seeding and enhanced their growth and proliferation. Mussel-inspired electrospun NFs doped with silver nanoparticles were formulated for wound healing applications by GhavamiNejad et al. using the catechol redox chemistry [163]. The silver nanoparticles themselves have antibacterial properties, and their activity was found to be size- and shape-dependent and the degree of aggregation is also engaged. Due to the absence of an external reducing agent, the arising tissue toxicity was minimized. The presence of 1% nanoparticles showed optimized antibacterial action against different strains of bacteria. The fibers offered a rapid release of nanoparticles in the first 24 h and could sustain release for about five days, which was the main requirement for effective antibacterial action (Figure 7).

**Figure 8:**
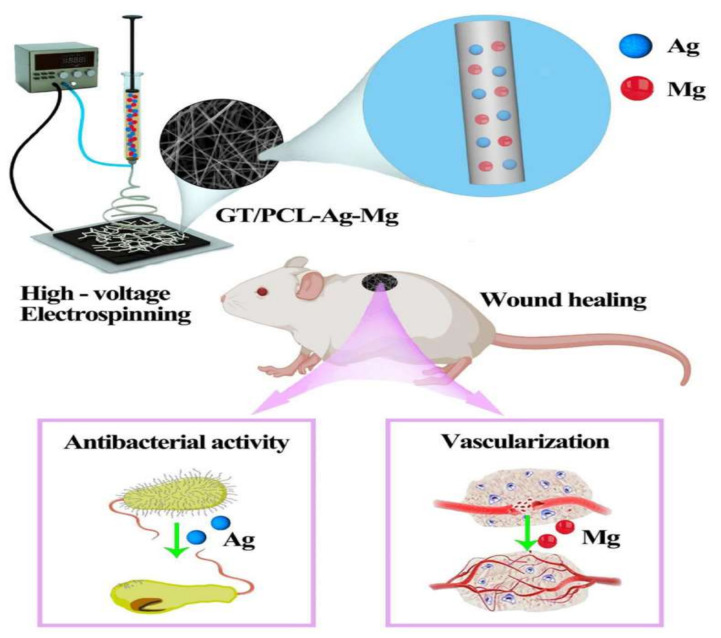
A gelatin (GT)/PCL NF membrane with Ag and Mg ions (GT/PCL-Ag-Mg) was fabricated, and its antibacterial and angiogenesis functions were demonstrated using in vitro and in vivo studies.

Similarly, other researchers developed wound dressing based on metallic ions such as silver and magnesium [164]. The wound dressing consisted of gelatin, PCL NFs loaded with metallic ions. The in vitro results show that the dressings showed good cytocompatibility and had good antibacterial activity against different bacterial strains. The presence of magnesium promoted the tube formation of vascular endothelial cells, epidermal formation, vascularization and collagen deposition (Figure 8).

Many attempts were made to encapsulate and deliver the natural extracts via electrospun NFs. Vargas et al. followed this approach using the *Calendula officinalis* and hyperbranched polyglycerol (HPGL) since the active ingredients had wound healing and anti-inflammatory action. The electrospun fibers showed rapid release owing to their high swelling ability and high porosity [165]. By increasing the *Calendula officinalis* concentration, a higher release rate from the fibers was observed. The in vivo experiments conducted on rats showed their potential wound healing characteristics.

## 3. Mechanical Properties of Electrospun NF Implants

The mechanical properties of electrospun nanofiber implants are critical in applications such as implants that require long-term endurance and structural integrity. To be useful in any application, fibers must have acceptable mechanical properties. Traditional tensile tests can be used to assess the electrospun nanofiber materials’ tensile strength. However, in most of the published research, these tests are performed on electrospun mats, scaffolds and other materials rather than the fiber itself because measuring these parameters for fibers in the nano or submicron range is very difficult [1,2].

During the service of electrospun nanofibers as implants, nanofibers are continuously subjected to stresses from the surrounding environment, which can significantly result in their permanent deformation. The mechanical limitations of electrospun nanofibers can hinder their use in implants and other applications. Despite of the high compatibility of gelatin fibers, they have poor mechanical properties (tensile strength, Young’s modulus, elongation, elasticity). Therefore, the applications and further development of gelatin-based nanofibers are compromised [3]. Similarly, PLA and PLGA-based scaffolds’ low mechanical properties resulted in their permanent deformation after a prolonged duration of cyclic strain [4]. On the other hand, the flexible mechanical proprieties of PCL-based nanofiber membranes resulted in their extensive study in various applications [5].

As a result, many approaches have been proposed to enhance the mechanical properties of electrospun nanofibers. Polymer blending is one of the used approaches to provide mechanically competent electrospun nanofiber-based implants. The tensile modulus of PCL polymer is 2.05 ± 0.15 MPa, which is much higher than the stiffness range reported for human myocardium in many literatures (20–50 KPa) [6]. Accordingly, the electrospun PCL-based implant does not successfully imitate the host ECM and thusdoes not support effective implant regeneration and proliferation into the myocardium. Castilho and colleagues created electrospun nanofibers made of miscible blend of PCL and poly(hydroxymethylglycolide-co-caprolactone) (pHMGCL) in various ratios to overcome the failure of PCL-based myocardial implant in cardiac tissue engineering [7]. The tensile modulus of a blend of pHMGCL and PCL (40:60) decreased from 2.05 ± 0.15 MPa to 1.18 ± 0.08 MPa, bringing it closer to the range of normal myocardium stiffness. Other approaches can be also used to provide mechanically competent electrospun nanofibers, such as the modification of the polymer structure and the modification of the processing method.

## 4. Portable ES Device Applications

Introducing portable ES devices to the field of nanomedicine has emerged, primarily to overcome possible drawbacks associated with conventional ES setups that usually consist of mega-sized equipment that is difficult to be assembled for instant applications in a one-time use manner. The mobility of this device and its deposited fibers have further expanded the promise of in situ electrospinning for applications in wound healing and other fields. Only the spinneret component of these devices is portable since the rest of the machine is too huge and heavy to be hand handled, as with most other portable gadgets. In addition, the resulting matrices are bulky with possible inadequate stability restricting their ease of transportation and storage, accounting for a higher risk of degradation. Portable ES devices, on the other hand, are easily assembled using completely available off-shelf materials while producing highly efficient fibers for point of need application [188], mainly implemented in wound dressings with skin regenerating qualities [189,190,191] and rapid hemostasis [192] either in minimally invasive surgeries [193] or outdoor settings [194].

**Figure 9:**
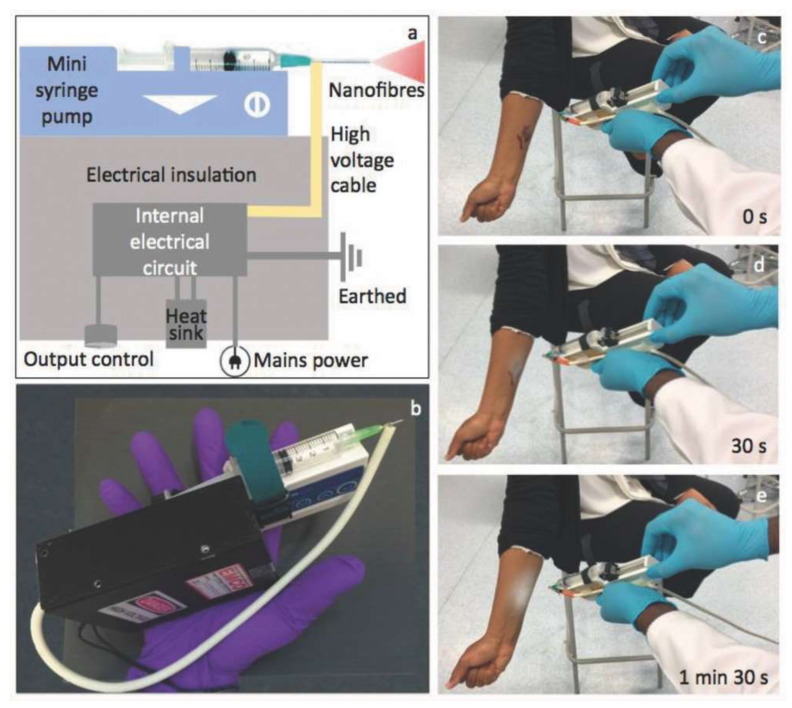
(**a**) Schematic diagram of the portable EHD device. (**b**) A photograph showing the assembly of the mini device held in hand. (**c**–**e**) Snapshots of real-time recording of the operation of the mini EHD device generating nanofibers onto a mock wound in situ [188] (Reproduced CC BY license).

Brako et al. [188] presented a simple, portable electrospinning device to overcome this constraint and the high cost of the equipment that was previously used to address it. This consists of a miniaturized high-precision microsyringe pump (Micrel mph+) as well as a minuscule high-voltage power supply (EMCO 4330+) that is capable of producing up to 33 kV at a power of 10 W (as shown in Figure 9). By completely combining the spinneret and the power supply unit into a portable device, the whole apparatus becomes lighter in weight and smaller, allowing it to be deployed almost everywhere where there is mains electricity. In this technology, CAc and CAc doped with silver nanoparticles have been electrospun into fibers that have been effectively applied directly to simulated wound sites. Such portable devices may point the way to the more widespread and practical use of functional nanofibers in advanced wound care that is more individualized.

Long and colleagues developed a simple portable battery-operated electrospinning apparatus (BOEA) that uses a finger-pressed syringe to generate electrospinning [195]. This device’s high-voltage supply was provided by a combination of batteries and a high-voltage converter. It has been stated that the charge may be passed via the body (hand) by contacting the metal foil, hence preventing charge buildup from occurring. It was found that two AAA batteries (3 V) were sufficient for the practical use of the BOEA and that it was capable of continuous operation for more than 15 h with insignificant current and an effective working distance ranging from 2 to 10 cm with little current consumption. One benefit to note is using a battery and a high-voltage converter; thus, the device was lightweight (about 120 g) and had small overall dimensions, thus enhancing its portability. Several polymers, including polyvinyl pyrrolidone, polyvinylidene fluoride and PCL, were successfully electrospun into fibers with diameters in the hundreds of nanometers range using the BOEA, which was also tested for solution electrospinning performance.

### 4.1. Wound Dressings with Skin Regenerating Properties

Edirisinghe and colleagues suggested a portable electrospinning system, including a portable handheld spinneret, to get in situ electrospinning results. Essentially, the instrument consisted of a spray pistol equipped with either a single needle or two coaxial needles. This device was used to create PLGA particles and fibers and PLGA/Polymethysilsesquioxane core-shell fibers deposited onto a limited target region at various angles [196]. Following that, the portable electrospinning apparatus was used for in situ deposition of bioproducts throughout the wound healing process, and the results were promising. In this case, the portable device offered a handy way of depositing PLGA fibers onto a target location, such as an incised wound or the surface of a grazed wound. Furthermore, due to the electrostatic attraction forces, fibers may be sprayed onto a burn wound site in less than 300 s, resulting in a thin protective coating on the surface of the burn wound. A simple hand flexing might be used to remove the film [197].

**Figure 10:**
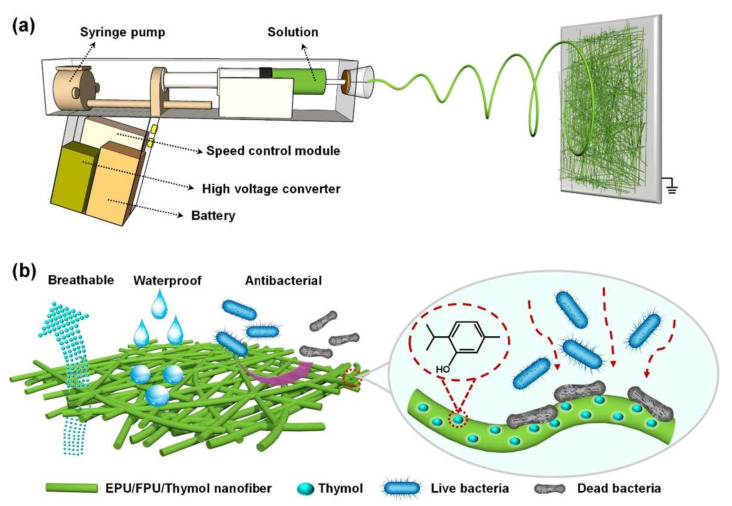
(**a**) Fabrication of EPU/FPU/Thymol nanofibrous membranes using the ES portable device. (**b**) Demonstrative scheme of the breathable, waterproof and antibacterial action of EPU/FPU/Thymol nanofibers. Republished under the permission of Yue et al. [154].

Yue et al., 2021 introduced an enhanced portable ES device matching the shape of the gun and imports an attached battery (3 V), voltage transformer module (from 3 V to 5 V), high voltage converter (amplifying the 5 V up to 11 kV), stepper motor syringe pump, and a speed control module allowing for high voltage supply and controllable perfusion speed (from 0.05 mL/h to 10 mL/h) to prepare waterproof and breathable (W&D) fiber matrices. In addition, the current values were much smaller than the perception current, the minimum current value (of about 1 mA) that was perceivable by the human body senses, that they were ignored and ruled as safe for human application [154]. They used ethanol-soluble polyurethane (EPU), fluorinated polyurethane (FPU) and thymol (2-isopropyl-5-methylphenol) to prepare the polymer solution, as shown in Figure 10. EPU/FPU formed the constructive basis of the fiber-matrix. At the same time, thymol added an antibacterial property to the generated skin-like matrices that were tuned precisely for every wound structure with excellent mechanical properties, pertaining to its porosity, thickness and moisture permeability with a high water vapor transmittance (WVT) rate of about 13.1 Kg m^−2^d^−1^, robust hydrophobicity with a moderate hydrostatic pressure of 17.6 cm H_2_O and excellent breathability of 3.56 kg m^−2^d^−1^ allowing for an overall enhanced patient experience [198].

Other possible candidates for the electrospun polymer solution used for preparing wound dressings include, but are not limited to, zein/thyme essential oil (TEO) [199], zein/clove essential oil (CEO) [200], zein and cinnamon oil [201] and PLA/gelatin [202]. The essential oil in every combination has antibacterial activity, and some even are active against Escherichia coli and Staphylococcus aureus, as in cinnamon oil with an inhibition zone of around 5 cm.

### 4.2. Rapid Hemostasis

**Figure 11:**
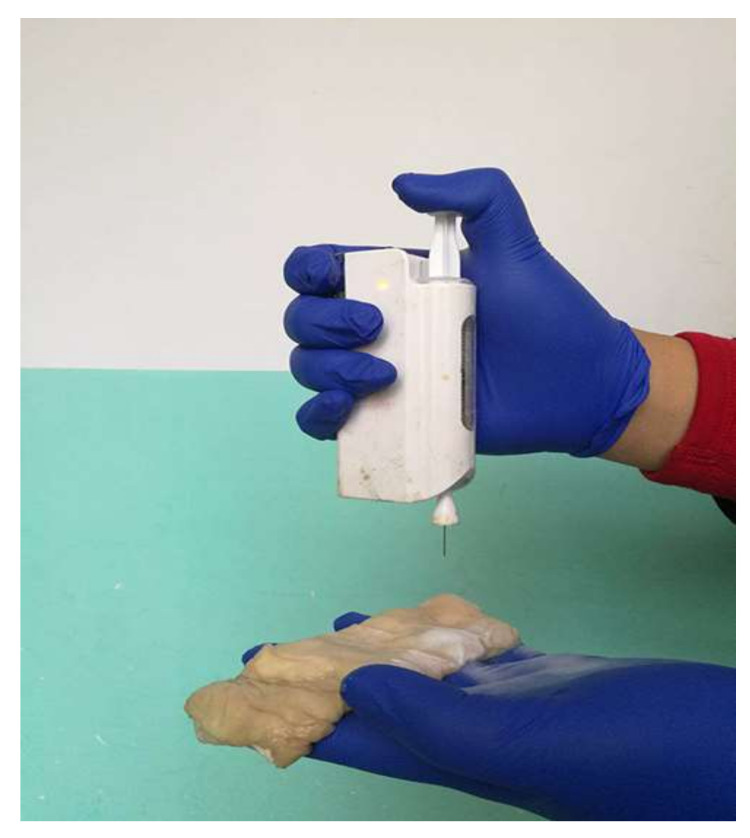
Implementing the portable ES device in an intestinal incision. With permission from ref. [192].

The application of conventional ES fibers in internal organs such as the liver and meningeal closures requires their fixation and the fibers to be thick enough to mediate its adhesion to the organ lining; however, this limits its use in sensitive wounds such as in visceral bleeding. Among the proposed materials, PCL appears to be the most convenient for visceral application superior to chitosan, alginate and cyanoacrylates (CA) [203]. Zhou et al., 2020 proposed that it is was possible to use a portable ES device for in situ simultaneous application of PCL ES fibers in rapid intestinal hemostasis, as shown in Figure 11. The generated fiber matrices had strong adsorptive forces to wound exudates and were able to resist hydrostatic pressure; additionally, they had an overall good sealing effect [192].

Laparoscopic aided ES was introduced by Zhang et al., 2020 when they applied a handheld ES device with a long needle (40 cm long, 0.5 cm in diameter) inserted in a laparoscopic tube (0.6 cm in diameter) during a minimally invasive operation (MIO) on a pig liver. This technique showed rapid hemostasis (5 s) consistent with reduced inflammatory responses and accelerated recovery rates [193]. However, safety issues are still a concern, and clinical trials should evaluate the extent to which a portable ES device is applied within critical conditions, as in surgeries, instead of sutures and other conventional techniques.

**Figure 12:**
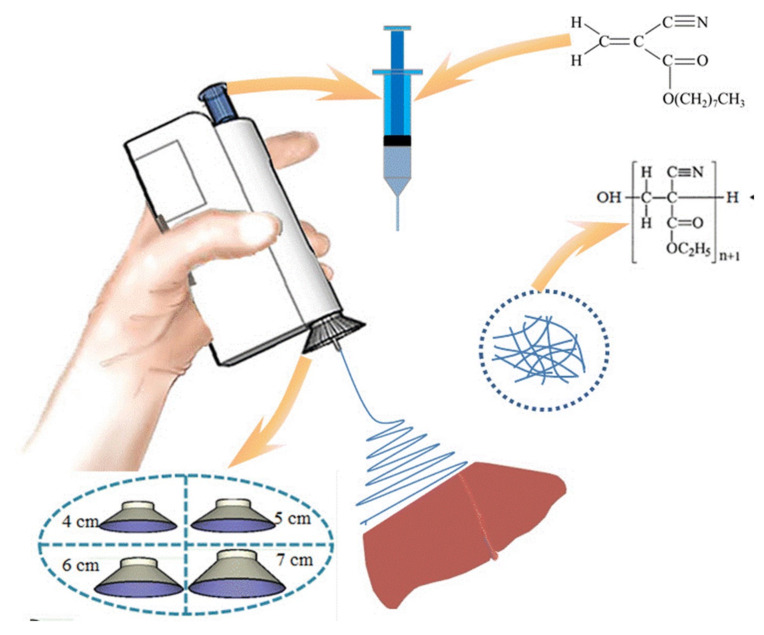
Schematic diagram of the electric field-modified e-spinning NOCA fibers for liver resection hemostasis.

The use of the BOEA for direct electrospinning of the medical adhesive, N-octyl-2-cyanoacrylate (NOCA), to create fibrous media suitable for soft tissue hemostasis, which is related to liver hemostasis, has been investigated more recently by the Luo et al. [204]. Controlling tissue adhesion after surgery is critical in this specific application, and accurate deposition of the hemostatic medical adhesive is often required to achieve this goal. It was necessary to attach a metal cone to the spinning nozzle to achieve precise deposition (as shown in Figure 12). It was discovered that by varying the metal cone’s size, the electrospun fibers’ deposition range could be modified to the desired value. Further, this modified BOEA was used to deposit NOCA fibers onto the resection site of the rat liver, resulting in rapid hemostasis within 10 s. When comparing the outcomes of this procedure to those of the airflow-assisted group, postoperative pathological findings revealed that the inflammatory response and tissue adhesion were decreased. A modified BOEA might be designed for emergency medical operations, community patient care or home care scenarios where the mobility and simplicity of operation would be especially helpful, as shown by this possibility. In this device, the voltage was fixed, limiting the extent to which the physical properties of dressings and the dimensions of dressings produced in situ could be modified. Additionally, the delivery of the spinning solution was dependent on the pressure applied by the fingers to the trigger, which could vary during use, resulting in inconsistent fiber deposition.

#### Future Perspectives

The recently introduced portable devices have increasingly superseded the usage of homemade electrospinning equipment with portable spinnerets in recent years. These devices are comprised of a spinneret in the form of a pistol, and an airflow became commercially available. Therefore, the airflow constrains the electrospinning jets, resulting in the exact placement of electrospun fibers on the target region. Furthermore, the interest in using in situ fiber in mildly invasive surgeries over the conventional methods is increasing. However, this technology is in the early phase of its applications in clinical studies [192]. The problem with the approach is that the human body’s conductive nature could precipitate short circuits and creepage. Therefore, further studies are necessary to establish cost-effective and less detrimental techniques required to meet the market demand. Besides that, in wound dressings level, rapid hemostasis, or even minimally invasive surgeries, fast, safe, and effective techniques are essential.

## 5. Conclusions

Advances in all biomedical domains and the increasing complexity of diagnosis and therapeutic processes necessitate the development of a unique drug delivery system to improve the bioavailability of pharmaceuticals and their actions, as well as their safety and adverse effects. Nanofiber biomaterials material fabricated by electrospinning possess extremely high specific surface area, high porosity, adjustable fiber morphology and surface function. Due to these properties, the electrospun fiber material can meet the requirements for advanced biomedicine and tissue engineering. Specifically, this paper aimed to explore the efficacy of electrospun nanofibers for application in wound treatment, antibacterial coating to prevent implant-related infections in orthopedic and dental applications, and implants for cancer therapy, by updating with information collected from the most recent literature. The ES of biopolymers and APIs is also discussed regarding their current scope, limits and future outlook. In the context of a nanotechnological approach for novel wound dressing fabrication, reducing scar formation and drug-loading with efficient therapeutic agents, this review may offer new perspectives and technical support for the development of advanced, functional, soft biomaterials from lab-scale to industrial-scale production.

## Figures and Tables

**Table 1:** Multidrug-loaded electrospun NF implants for cancer treatment.

Polymeric Fiber	ES Technique	Drugs	Type of Cancer	In Vivo	In Vitro	Ref.
BIC/(PLGA)	Blend ES	CAR, CIS and CPT-11	Brain cancer (C6 glioma)	+		[43]
	Blend ES	5-FU and oxaliplatin	Colorectal cancer (HCT8 and CT26 cell lines)	+	+	[44]
PGC-C18/PCL	Blend ES	CPT-11 and SN-38	Human colorectal (HT-29) cell line		+	[45]
PLGA/gelatin	Blend ES	DOX-encapsulated mesoporous zinc oxide microspheres /camptothecin	Liver cancer (HepG2) cell line		+	[46]
PEG/PLA	Blend ES	CBT A-4 and HCPT	Breast (4T1) tumor model	+	+	[47]
	Emulsion ES	Paclitaxel and DOX	Brain cancer (C6 glioma)		+	[48]
Dextran/PLGA	Emulsion ES	HCPT and tea polyphenol	Orthotopic liver (H22) carcinoma cell line			[49]
PCL/gelatin	Second carrier ES (core/shell silica nanoparticles)	DOX and Indomethacin	L929 fibroblast cells			[50]
PLGA	Sequential ES	CAR, CPT-11, CIS and CBT	Brain cancer (C6 glioma)	+		[51]
	Emulsion ES	Paclitaxel and Brefeldin A	Human liver (HepG2) cancer cell line		+	[52]
PLLA	Sequential ES	DCA and oxaliplatin	Cervical cancer (Hela cancer and U14 cancer cell lines)	+	+	[53]
	Second carrier ES (mesoporous silica nanoparticles)	DOX/Ibuprofen	Cervical cancer (Hela cell line)		+	[54]
	Blend ES	DOX and HCPT	Human cervical cancer (HeLa cells)		+	[55]
		DCA and diisopropylamine dichloroacetate	Colorectal cancer (C26 cells)	+	+	[56]
		Oxaliplatin and cyclophosphamide	Human hepatocellular cancer (HCC cells)	+	+	[57]
PCL	Blend ES	(−)-epigallocatechin-3-O-gallate and caffeic acid	Human gastric cancer MKN28 cells		+	[58]
		Cisplatin and CUR	Human cervical cancer (HeLa cells)	+	+	[59]
		CUR and aloe-vera or neem-extract	Lung carcinoma (A549) and breast cancer (MCF-7)		+	[60]
	Core-sheath ES	Ibuprofen and DOX	Human hepatocellular carcinoma cell line (HuH-7)		+	[61]
		5-FU and paclitaxel	TNBC cells human triple-negative breast cancer	+	+	[62]
PLLA/PCL	Microfluidic ES	DOX and angiogenesis inhibitor apatinib	Breast cancer (4T1 cells)	+	+	[63]
PVA	Second carrier ES (mPEG-PCL micelles)	DOX and CUR	Cervical cancer (Hela) cell line		+	[64]
	Blend ES	Dichloroacetate and Pt(IV) prodrug-backboned micelle	HeLa human cervical cancer cells	+	+	[65]
5-FU: 5-fluorouracil; CAR: Carmustine; CBT: combretastatin; CIS: Cisplatin; CPT-11: irinotecan; CUR: Curcumin; DCA: Sodium dichloroacetate; DOX: Doxorubicin; HCPT: Hydroxycamptothecin; PGC-C18: Poly(glycerol monostearate-co-ε-caprolactone); PLLA: Poly (l-lactic acid); PLGA: (D, L-lactic acid-co-glycolic acid); and SN-38: irinotecan metabolite.						

**Table 2:** Therapeutic agents loaded into electrospun NF implants to promote wound healing.

Polymeric Fiber	Therapeutic Agent	Purpose	Ref.
PLLA/PVA	Cefazoline	Antibacterial	[97]
PLGA			[98]
Chitosan/PVA	Lysozyme	Antimicrobial	[99]
PCL	Chloramphenicol	Antibacterial	[100]
PLGA	Quercetin	Cell proliferation and adhesion/antibacterial	[101]
PCL	Rifampicin	Antimicrobial	[102]
PCL/Gelatin	Metronidazole	Antibacterial	[81]
PCL	Tauroursodeoxycholic acid	Angiogenesis	[103]

**Table 3:** Electrospun NF coatings for implant-related infections.

Polymeric Fiber	Antibacterial Agent	Implant	Antibacterial Action against	Ref.
PCL/HA (Polymer/Ceramic)	Rifampicin	Titanium (orthopedic)	*Staphylococcus aureus*, *Staphylococcus epidermidis*, *Pseudomonas aeruginosa* and *MRSA*	[133]
PLGA/PCL	Vancomycin and Rifampicin	Titanium (orthopedic)	*Staphylococcus aureus*	[137]
PLGA/PCL	Vancomycin/Rifampicin Linezolid/rifampicin Daptomycin/rifampicin	Titanium (orthopedic)	*MRSA*	[138]
PLGA	Vancomycin	Titanium (orthopedic)	*Staphylococcus aureus*	[132]
PLA/PCL/gelatin	Tetracycline	Titanium (dental)	*Prevotella intermedia*, *Porphyromonas gingivalis*, *Fusobacterium nucleatum* and *Porphyromonas gingivalis*	[141]
Chitosan/polyethylene oxide (PEO)	Vancomycin	Titanium (orthopedic)	*Staphylococcus aureus*	[142]
Keratin	Silver	Titanium (dental)	*Staphylococcus aureus*	[143]
PCL/PVA	Doxycycline	Titanium (orthopedic)	*Staphylococcus aureus*	[144]
Carboxymethylcellulose (CMC)/ PEO	Clindamycin	AISI 316LVM (stainless steel) and Ti90Al6V4 (alloy) (orgopedic)	Staphylococci, streptococci, pneumococci, and bacteroides species	[145]
PLGA/PEO	Gentamycin	Titanium (orthopedic)	*Staphylococcus aureus*	[146]

**Table 4:** A literature review of various antibacterial wound dressings prepared using the ES technique.

Polymeric Chain	Active Agents	Antibacterial Action against	Ref.
PLA/PCL	Tetracycline hydrochloride	*Staphylococcus aureus*, *Escherichia coli* and *Pseudomonas aeruginosa*	[166]
PLGA	Cefoxitin sodium	*Staphylococcus aureus*	[167]
	Amoxicillin	*Staphylococcus aureus*	[168]
PLA	Mupirocin	*Staphylococcus aureus*	[169]
coPLA/PEG	Ciprofloxacin hydrochloride, levofloxacin hemihydrate or moxifloxacin hydrochloride	*Staphylococcus aureus*	[170]
PLA, PLA/Collagen	Gentamicin	*Escherichia coli*, *Staphylococcus epidermidis*, *Pseudomonas aeruginosa*	[171]
PLLACL	Tetracycline hydrochloride	*Escherichia coli*	[172]
PMMA/Nylon 6	Ampicillin	*Listeria innocua*	[173]
PAA	Doxycycline hyclate	*Staphylococcus aureus*, *Streptococcus agalactiae*, *Pseudomonas aeruginosa*	[174]
Cyclodextrin complex	Triclosan	*Staphylococcus aureus*, *Escherichia coli*	[175]
PCL/PLA	N-halamine	*Escherichia coli*, *Staphylococcus epidermidis*	[176]
PLA		*Staphylococcus aureus*, *Escherichia coli*	[177]
PAN		*Staphylococcus aureus*, *Escherichia coli*	[178]
CAc	Quaternary ammonium salts	*Staphylococcus aureus*, *Escherichia coli*	[179]
	Chlorhexidine	*Escherichia coli*, *Staphylococcus epidermidis*	[180]
CAc/PEU	Polyhexamethylene biguanide	*Escherichia coli*	[181]
PEO/Chitosan	Potassium 5-nitro-8-quinolinolate	*Staphylococcus aureus*, *Escherichia coli*, *Candida albicans*	[182]
PAN	Silver NPs	*Staphylococcus aureus*, *Escherichia coli*, *Bacillus subtilis*	[183]
PLA/Chitosan		*Staphylococcus aureus*, *Escherichia coli*	[184]
PEO/Chitosan	Antimicrobial peptides (Plantaricin 423 and bacteriocin ST4SA)	*Escherichia coli*	[185]
PVA/Chitosan		*Escherichia coli*	[186]
PDLLA/PEO		*Enterococcus faecium*	[187]

Abbreviations. CAc: Cellulose acetate; coPLA: poly(l-lactide-co-d,l-lactide); PAA: poly(acrylic acid); PAN: polyacrylonitrile; PEU: polyester urethan; PCL: poly(ϵ-caprolactone); PDLLA: poly(d,l-lactide); PEG: polyethylene glycol; PEO: poly(ethylene oxide); PLA: poly(lactic acid); PLGA: poly(lactide-co-glycolide); PLLACL: poly(l-lactid-co-ε-caprolactone); PMMA: poly(methyl methacrylate); and PVA: polyvinyl alcohol.

## Data Availability

Not applicable.
